# Social determinants of health and their associations with outcomes in pediatric out-of-hospital cardiac arrest: A national study of the NEMSIS database

**DOI:** 10.1016/j.resplu.2024.100795

**Published:** 2024-10-04

**Authors:** Mary E. Bernardin, Jyoti Arora, Paul Schuler, Benjamin Fisher, Joseph Finney, Elizabeth Kendrick, Danielle Lee

**Affiliations:** a Division of Pediatric Emergency Medicine, Department of Emergency Medicine, University of Missouri School of Medicine, 1 Hospital Drive, Columbia, MO 65212, United States; b Division of Pediatric Emergency Medicine, Department of Pediatrics, Washington University in St. Louis School of Medicine, 1 Children’s Place, St. Louis, MO 63110, United States; c Centre for Biostatistics and Data Science, Washington University School of Medicine, 660 S Euclid Ave, St. Louis, MO 63110, United States; d Division of Research, Department of Emergency Medicine, University of Missouri School of Medicine, 1 Hospital Drive, Columbia, MO 65212, United States; e Department of Pediatrics, University of Utah School of Medicine, 295 Chipeta Way, PO Box 581289, Salt Lake City, UT 84158, United States

**Keywords:** Social determinants of health (SDOH), Pediatric out-of-hospital cardiac arrest (POHCA), Bystander cardiopulmonary resuscitation (CPR), Automated external defibrillator (AED) usage, Return of spontaneous circulation (ROSC), Health disparities, Public health

## Abstract

**Background:**

Social determinants of health (SDOH) impact health disparities, though little is known about the effects of SDOH on pediatric out-of-hospital cardiac arrest (POHCA).

**Methods:**

This cross-sectional study utilized the NEMSIS Database to obtain nationwide POHCA data from 2021 to 2023. Outcomes included performance of bystander cardiopulmonary resuscitation (CPR), automated external defibrillator (AED) usage, and obtainment of return of spontaneous circulation (ROSC). SDOH data was obtained from the US Census Bureau and included minority race/ethnicities status, poverty levels, and educational attainment of the community where POCHAs occurred. Multivariable logistic regression and Cochran-Armitage trend tests were used to assess associations between SDOH and POHCA outcomes.

**Results:**

Query of the NEMSIS Database yielded 27,137 POHCAs. The odds of CPR performance and obtainment of ROSC were significantly higher (p < 0.001) in communities with lower levels of minority races/ethnicities. The odds of bystander CPR, AED usage, and obtainment of ROSC all increased significantly (p < 0.001) in the wealthiest communities compared to the poorest communities. The odds of bystander AED usage (p = 0.001) and ROSC (p = 0.003) were significantly higher in communities with the highest educational attainment. As the minority status and poverty level of the community increased and educational attainment decreased, there was a significant decreasing trend (p < 0.001) in performance of bystander CPR, AED usage, and obtainment of ROSC.

**Conclusions:**

Community-level SDOH, including increasing community minority status, poverty levels, and decreasing educational attainment, are associated with less bystander CPR, AED usage, and ROSC obtainment in POHCAs. Understanding SDOH offers opportunities for public health interventions addressing disparities in POHCA outcomes.

## Highlights

• Social determinants of health are related to pediatric cardiac arrest outcomes.
• Increased minority and poverty levels are associated with poorer outcomes.
• Higher community education levels are associated with better outcomes.
• Addressing health disparities requires understanding social determinants.

## Introduction

Pediatric out-of-hospital cardiac arrest (POHCA), or the loss of a child’s cardiac function and effective circulation in an out-of-hospital setting, is an austere event with only 6–9 % of children surviving to hospital discharge.^1^, ^2^ While POHCA is relatively infrequent, occurring in 1–20/100,000,^2^, ^3^, ^4^ the impact on a child’s life, family, and community are immeasurable.

Social determinates of health (SDOH) are the external conditions in which an individual lives, such as a person’s socioeconomic status or educational opportunities, which impact health outcomes and can contribute greatly to health disparities.^5^ In adult populations, a multitude of studies have described associations between adverse SDOH and outcomes relating to out-of-hospital cardiac arrest (OHCA).^6^, ^7^ Most notably, socioeconomic status has consistently been linked to increased incidence of OHCA, as well as lower automated external defibrillator (AED) usage and lower occurrence of cardiopulmonary resuscitation (CPR) attempted by bystanders.^7^ Understanding SDOH as they relate to OHCA is not only imperative for identifying those most at risk for poor outcomes, but is essential for identifying modifiable factors (e.g. lack of CPR or AED training) such that targeted interventions can be implemented and health disparities can be addressed.

Far fewer studies have evaluated impacts of SDOH on children experiencing POHCA, and those that have offer conflicting results.^1^ Some studies have reported lower POHCA survival amongst children of minority race/ethnicities,^8^, ^9^ whereas others have found no significant association.^10^, ^11^, ^12^, ^13^ Additional studies have reported lower rates of bystander CPR^8^, ^14^ and AED usage^15^ amongst children of minority race/ethnicity^8^, ^14^ or lower socioeconomic status,^15^, though others have found no such relationship.^9^, ^16^, ^17^, ^18^, ^19^ A consistent shortcoming of these studies and possible explanation for inconsistent findings is small sample sizes, with many studying under 200 children.^9^, ^11^, ^17^, ^20^, ^21^, ^22^, ^23^, ^24^

The objective of our study was to utilize the National Emergency Medical Services Information System (NEMSIS) Database to ascertain from an expansive, nationwide sample if SDOH, such as community racial/ethnic composition, poverty levels, and educational attainment, are associated with outcomes relating to POHCA, namely performance of bystander CPR, utilization of AEDs prior to EMS arrival, and obtainment of return of spontaneous circulation (ROSC).

## Methods

### Data collection and definitions

In this retrospective cross-sectional study, we utilized the EMS Data Cube to obtain national data from the NEMSIS Database on POHCA of all causes. The NEMSIS Database was established by the National Highway Traffic Safety Administration (NHTSA) and is a repository of data voluntarily reported from over 14,000 EMS agencies serving 54 states and territories in the United States (US).^25^, ^26^ EMS Data Cube Version 3 is a publicly available analytic tool which contains nationwide NEMSIS data from the previous 2 years to the calendar year to date.^27^ We queried the EMS Data Cube and obtained nationwide POHCA data from January 2021 through November 2023. The Washington University School of Medicine Institutional Review Board approved this study.

Our query was limited to EMS activations for out-of-hospital cardiac arrests occurring prior to EMS arrival in children under 18 years of age. All POHCA etiologies, both traumatic and non-traumatic, were included. Individuals with multiple encounters for POHCA or missing the following data elements were excluded. Data elements obtained included patient age in years, performance of bystander CPR prior to EMS arrival, the application of an AED with and without defibrillation prior to EMS arrival, and the obtainment of ROSC both before and after arrival at an emergency department.

In order to assess for associations between SDOH and POHCA outcomes, we obtained NEMSIS information regarding minority race/ethnicity status, poverty levels, and educational attainment of individuals in the community in which the POHCA occurred. This data was obtained directly from the NEMSIS EMS Data Cube, which pulls US Census Bureau statistics from Census.gov^28^ Minority race/ethnicity status is reported in percentage categories < 5 %, 6 %-10 %, 11 %-25 %, 26–50 %, and ≥ 51 %. These US Census Bureau statistics classifies minority status as the percentage of residents in a given zip code that do not self-identify as “white.” “Minority” race/ethnicities thus include but are not limited to Black or African American individuals, American Indian or Alaska Native individuals including persons with origins in any of the original peoples of North America, South America, Central America, Asian Americans, Native Hawaiian or other Pacific Islander individuals.^28^

Community poverty level is reported in percentage categories < 5 %, 6 %-10 %, 11 %-15 %, 16–20 %, 21 %-25 %, and ≥ 26 %. These statistics are obtained from the US Census Bureau, which defines the US Census Poverty Percent as the percentage of residents in a given zip code that are living in households with income below the federal poverty level based on the 2014 American Community Survey.^28^

Community educational attainment is reported in percentage categories < 70 %, 71 %-75 %, 76 %-80 %, 81–85 %, 86 %-90 %,91 %-95 %, and 96 %-100 %. These statistics are obtained from the US Census Bureau which defines the US Education Percent as the percentage of residents in a given zip code that have completed high school or higher education.^28^

### Data analysis

Continuous variables are summarized as means with standard deviations, and categorical variables as frequencies and percentages. Multivariable logistic regression was used to assess for associations between community minority status, poverty, and educational attainment and odds of bystander CPR performance, use of an AED, and obtainment of ROSC. To account for potential confounding, we adjusted for patient age and sex. To perform the multivariable analysis using logistic regression, we assumed the independence of observations, the linearity between the logit of the dependent variables and the continuous independent variables, absence of substantial multicollinearity between the independent variables, and adequate sample size. We computed variance inflation factors to check for multicollinearity and used Box-Tidwell test to examine the linearity assumption. The assumption of linearity was found to be not met between the outcome of AED usage and age and was addressed by using the link function of generalized logit for the analysis. All other assumptions were not violated. Odds ratios (OR) with 95 % confidence intervals (95 %CI) and p-values are provided to describe the magnitude and significance of the associations. Cochran-Armitage trend tests were used to assess for statistically significant trends between community minority status, poverty, and educational attainment as they relate to performance of CPR, AED usage, and obtainment of ROSC. P values < 0.05 were considered statistically significant. Statistical analyses were performed using Python software, version 3.12.5,^29^ and SAS software, version 9.4.^30^

Query of the NEMSIS Database resulted in 27,137 POHCAs that met inclusion criteria. Child demographics and POHCA supplemental data can be found in Table 1. Ages ranged from less than 1 year to a maximum age of 17 years with a mean age of 5.51 years (standard deviation of 6.43 years). The majority of children were male (58 %) and White/Caucasian (38.2 %). The most frequent presumed arrest etiology was traumatic (29.5 %). POHCAs were most frequently unwitnessed (59.7 %) and in asystole (62.3 %) upon EMS arrival. ROSC was obtained in 5,624 cases, or 20.7 % of POHCA occurrences. Bystander CPR was performed in 16,774 cases (61.8 %), including 73.4 % of the cases in which ROSC was obtained. Bystander AEDs application occurred in 6,209 cases (22.9 %), including 26.1 % of the cases in which ROSC was obtained.

**Table 1:** Demographic and POHCA supplemental data.

**Variables**	**All**	**Bystander CPR Only**	**AED Use Only**	**Bystander CPR and AED Use**	**No Interventions**	**ROSC Obtained**	**No ROSC**
**N**	27,137	11,774 (43.39 %)	1,209 (4.46 %)	5,000 (18.43 %)	9,154 (33.73 %)	5,624 (20.72 %)	21,513 (79.28 %)
**Age, years**							
**0 to 2**	14,423 (53.15 %)	7,044 (59.83 %)	579 (47.89 %)	2,370 (47.40 %)	4,430 (48.39 %)	2,510 (44.63 %)	11,913 (55.38 %)
**3 to 5**	2,460 (9.07 %)	1,067 (9.06 %)	99 (8.19 %)	476 (9.52 %)	818 (8.94 %)	598 (10.63 %)	1,862 (8.66 %)
**6 to 11**	2,982 (10.99 %)	1,133 (9.62 %)	142 (11.75 %)	622 (12.44 %)	1,085 (11.85 %)	733 (13.03 %)	2,249 (10.45 %)
**12 to 17**	7,272 (26.80 %)	2,530 (21.49 %)	389 (32.18 %)	1,532 (30.64 %)	2,821 (30.82 %)	1,783 (31.70 %)	5,489 (25.51 %)
**Sex**							
**Female**	10,633 (39.18 %)	4,731 (40.18 %)	444 (36.72 %)	1,883 (37.66 %)	3,575 (39.05 %)	2,176 (38.69 %)	12,428 (57.77 %)
**Male**	15,731 (57.97 %)	6,748 (57.31 %)	714 (59.06 %)	2,970 (59.40 %)	5,299 (57.89 %)	3,303 (58.73 %)	8,457 (39.31 %)
**Not Recorded**	773 (2.85 %)	295 (2.51 %)	51 (4.22 %)	147 (2.94 %)	280 (3.06 %)	145 (2.58 %)	628 (2.92 %)
**Race/** **Ethnicity**							
**White/** **Caucasian**	10,362 (38.18 %)	4,760 (40.43 %)	429 (35.48 %)	2,195 (43.90 %)	2,978 (32.53 %)	2,323 (41.31 %)	8,039 (37.37 %)
**Black/** **African**	7,231 (26.65 %)	3,050 (25.90 %)	406 (33.58 %)	1,264 (25.28 %)	2,511 (27.43 %)	1,184 (21.05 %)	6,047 (28.11 %)
**Hispanic/** **Latino**	2,930 (10.80 %)	1,288 (10.94 %)	135 (11.17 %)	463 (9.26 %)	1,044 (11.40 %)	639 (11.36 %)	2,291 (10.65 %)
**Multi Race/** **Ethnicity**	1,003 (3.70 %)	453 (3.85 %)	60 (4.96 %)	235 (4.70 %)	255 (2.79 %)	436 (7.75 %)	567 (2.64 %)
**Asian**	422 (1.56 %)	204 (1.73 %)	15 (1.24 %)	91 (1.82 %)	112 (1.22 %)	108 (1.92 %)	314 (1.46 %)
**American Indian or Alaska**	205 (0.76 %)	104 (0.88 %)	7 (0.58 %)	27 (0.54 %)	67 (0.73 %)	44 (0.78 %)	159 (0.74 %)
**Native Hawaiian**	109 (0.40 %)	53 (0.45 %)	5 (0.41 %)	26 (0.52 %)	25 (0.27 %)	22 (0.39 %)	89 (0.41 %)
**Not Recorded**	4,875 (17.96 %)	1,862 (15.81 %)	152 (12.57 %)	699 (13.98 %)	2,162 (23.62 %)	868 (15.43 %)	4,007 (18.63 %)
**Initial Rhythm**							
**Asystole**	16,912 (62.32 %)	8,078 (68.61 %)	811 (67.08 %)	3,376 (67.52 %)	4,647 (50.76 %)	2,120 (37.70 %)	14,792 (68.76 %)
**PEA**	3,779 (13.93 %)	1,626 (13.81 %)	205 (16.96 %)	618 (12.36 %)	1,330 (14.53 %)	1,430 (25.43 %)	2,349 (10.92 %)
**Unknown Non-shockable**	1,661 (6.12 %)	640 (5.44 %)	120 (9.93 %)	537 (10.74 %)	364 (3.98 %)	686 (12.20 %)	975 (4.53 %)
**Unknown Shockable**	401 (1.48 %)	83 (0.70 %)	21 (1.74 %)	250 (5.00 %)	47 (0.51 %)	231 (4.11 %)	170 (0.79 %)
**Ventricular Fibrillation**	850 (3.13 %)	360 (3.06 %)	56 (4.63 %)	217 (4.34 %)	217 (2.37 %)	382 (6.79 %)	468 (2.18 %)
**Ventricular Tachycardia-Pulseless**	180 (0.66 %)	65 (0.55 %)	12 (0.99 %)	52 (1.04 %)	51 (0.56 %)	97 (1.72 %)	83 (0.39 %)
**Not Recorded**	4,526 (16.68 %)	1,441 (12.24 %)	55 (4.55 %)	214 (4.28 %)	2,816 (30.76 %)	1,196 (21.27 %)	3,330 (15.48 %)
**Presumed Arrest Etiology**							
**Cardiac**	7,543 (27.80 %)	3,335 (28.33 %)	376 (31.10 %)	1,750 (35.00 %)	2,082 (22.74 %)	1,313 (23.35 %)	6,230 (28.96 %)
**Drowning/** **Submersion**	2,180 (8.03 %)	1,273 (10.81 %)	66 (5.46 %)	455 (9.10 %)	386 (4.22 %)	894 (15.90 %)	1,286 (5.98 %)
**Drug Overdose**	728 (2.68 %)	317 (2.69 %)	42 (3.47 %)	136 (2.72 %)	233 (2.55 %)	306 (5.44 %)	422 (1.96 %)
**Electrocution**	27 (0.10 %)	3 (0.03 %)	1 (0.08 %)	11 (0.22 %)	12 (0.13 %)	15 (0.27 %)	12 (0.06 %)
**Exsanguination-Medical**	97 (0.36 %)	38 (0.32 %)	3 (0.25 %)	20 (0.40 %)	36 (0.39 %)	23 (0.41 %)	74 (0.34 %)
**Respiratory/** **Asphyxia**	4,964 (18.29 %)	1,478 (12.55 %)	157 (12.99 %)	572 (11.44 %)	2,757 (30.12 %)	551 (9.80 %)	4,413 (20.51 %)
**Traumatic Cause**	8,000 (29.48 %)	4,048 (34.38 %)	362 (29.94 %)	1,524 (30.48 %)	2,066 (22.57 %)	2,155 (38.32 %)	5,845 (27.17 %)
**Other**	4,170 (15.37 %)	1,491 (12.66 %)	250 (20.68 %)	702 (14.04 %)	1,727 (18.87 %)	762 (13.55 %)	3,408 (15.84 %)
**Not Recorded**	600 (2.21 %)	310 (2.63 %)	23 (1.90 %)	94 (1.88 %)	173 (1.89 %)	123 (2.19 %)	477 (2.22 %)
**Arrest Witnessed By**							
**Not Witnessed**	16,202 (59.70 %)	7,661 (65.07 %)	735 (60.79 %)	3,275 (65.50 %)	4,531 (49.50 %)	2,145 (38.14 %)	14,057 (65.34 %)
**Family Member**	5,641 (20.79 %)	2,941 (24.98 %)	259 (21.42 %)	1,079 (21.58 %)	1,362 (14.88 %)	2,126 (37.80 %)	3,515 (16.34 %)
**Healthcare Provider**	2,332 (8.59 %)	573 (4.87 %)	206 (17.04 %)	310 (6.20 %)	1,243 (13.58 %)	1,009 (17.94 %)	1,323 (6.15 %)
**Bystander**	1,827 (6.73 %)	826 (7.02 %)	68 (5.62 %)	508 (10.16 %)	425 (4.64 %)	711 (12.64 %)	1,116 (5.19 %)
**Not Recorded**	2,307 (8.50 %)	292 (2.48 %)	12 (0.99 %)	92 (1.84 %)	1,911 (20.88 %)	151 (2.68 %)	2,156 (10.02 %)

Results of multivariable logistic regression modeling and Cochran-Armitage trend tests are depicted in Table 2. Logistic regression modeling revealed that a community’s minority race/ethnicity prevalence had a significant inverse association with the odds of bystander CPR performance and obtainment of ROSC for POHCA. The odds of CPR performance and obtainment of ROSC were 22 % and 17 % higher, respectively, in communities with 11–25 % minority status compared to ≥ 51 % minority status [OR 1.22 (95 %CI 1.12–1.33); OR 1.17 (95 %CI 1.05–1.29); p < 0.001]. Cochran-Armitage trend tests revealed that as the minority race/ethnicity status of the community increased, there was a statistically significant decreasing trend in performance of bystander CPR (p = 0.0015), AED usage (p < 0.001), and obtainment of ROSC (p < 0.001) for POHCA.

**Table 2:** Multivariable logistic regression modelling and Cochran-Armitage trend tests of performance of CPR, usage of AED, and attainment of ROSC for POHCA by percentage of community minority race/ethnicities, poverty level, and educational attainment.

			**CPR**				**AED**				**ROSC**			
Covariate	Level	N	Odds Ratio (95 % CI)	OR P-value	Regression Type 3 P-value	Cochran-Armitage Trend Test P-value	Odds Ratio (95 % CI)	OR P-value	Regression Type 3 P-value	Cochran-Armitage Trend Test P-value	Odds Ratio (95 % CI)	OR P-value	Regression Type 3 P-value	Cochran-Armitage Trend Test P-value
**% Minority**	≤5%	4145	0.88 (0.80–0.97)	**0.009**	**<0.001**	**0.0015**	0.92 (0.82–1.03)	0.143	0.365	**<0.001**	0.98 (0.87–1.11)	0.771	**<0.001**	**<0.001**
	6 %-10 %	3310	1.17 (1.06–1.30)	**0.002**			1.02 (0.90–1.14)	0.792			1.07 (0.95–1.21)	0.278		
	11 %-25 %	8090	1.22 (1.12–1.33)	**<0.001**			0.96 (0.87–1.05)	0.370			1.17 (1.05–1.29)	**0.004**		
	26 %-50 %	6653	1.24 (1.14–1.34)	**<0.001**			0.99 (0.89–1.08)	0.764			1.21 (1.10–1.34)	**<0.001**		
	≥51 %*	4939	−	−			−	−			−	−		
**% Poverty**	≤5%	2385	1.98 (1.72–2.28)	**<0.001**	**<0.001**	**<0.001**	1.68 (1.44–1.96)	**<0.001**	**<0.001**	**<0.001**	1.55 (1.32–1.81)	**<0.001**	**<0.001**	**<0.001**
	6 %-10 %	5495	1.76 (1.58–1.96)	**<0.001**			1.39 (1.22–1.58)	**<0.001**			1.45 (1.27–1.66)	**<0.001**		
	11 %-15 %	5624	1.47 (1.33–1.63)	**<0.001**			1.18 (1.05–1.33)	**0.005**			1.29 (1.14–1.46)	**<0.001**		
	16 %-20 %	5360	1.16 (1.05–1.27)	**0.002**			1.07 (0.95–1.19)	0.271			1.08 (0.96–1.21)	0.204		
	21 %-25 %	3589	0.90 (0.82–0.99)	**0.027**			0.97 (0.86–1.09)	0.618			0.96 (0.85–1.09)	0.516		
	≥26 %*	4684	−	−			−	−			−	−		
**% Education**	96 %-100 %	1536	1.13 (0.94–1.35)	0.184	**<0.001**	**<0.001**	1.28 (1.05–1.56)	**0.016**	**0.001**	**<0.001**	1.27 (1.03–1.55)	**0.023**	**0.003**	**<0.001**
	91 %-95 %	6913	0.82 (0.72–0.94)	**0.004**			1.16 (0.99–1.36)	0.074			1.00 (0.85–1.18)	0.957		
	86 %-90 %	7180	0.84 (0.74–0.95)	**0.006**			1.16 (0.99–1.35)	0.059			0.99 (0.84–1.14)	0.855		
	81 %-85 %	5166	0.97 (0.86–1.10)	0.669			1.26 (1.09–1.47)	**0.003**			0.99 (0.85–1.15)	0.872		
	76 %-80 %	3090	1.07 (0.94–1.21)	0.328			1.34 (1.14–1.57)	**<0.001**			1.14 (0.97–1.34)	0.116		
	71 %-75 %	1607	0.95 (0.82–1.09)	0.468			1.09 (0.91–1.30)	0.379			1.09 (0.90–1.30)	0.380		
	≤70 %*	1645	−	−			−	−			−	−		

OR = Odds ratio.

CI = Confidence interval.

* = Reference level.

Multivariable logistic regression modeling revealed that community poverty levels had a significant inverse association with odds of performance of bystander CPR, usage of AED, and obtainment or ROSC for POHCAs. The odds of bystander CPR performance increased by 98 % [OR 1.98 (95 %CI 1.72–2.28); p < 0.001] and odds of ROSC increased by 55 % [OR 1.55 (95 %CI 1.32–1.81); p < 0.001] in the wealthiest communities compared to communities with highest poverty levels. Cochran-Armitage trend tests confirmed that as community poverty increased, there was a statistically significant decreasing trend in performance of bystander CPR (p < 0.001), AED usage (p < 0.001), and obtainment of ROSC (p < 0.001) for POHCA.

Multivariable logistic regression modeling revealed that community educational attainment was significantly associated with performance of bystander AED usage and obtainment of ROSC for POHCAs. The odds of bystander AED usage [OR 1.28 (95 %CI 1.05–1.56); p = 0.02] and obtainment of ROSC [OR 1.27 (95 %CI 1.03–1.55); p = 0.02] increased by 28 % and 27 %, respectively, in the most educated communities compared to communities with the lowest educational attainment. Prior to adjusting for patient age and sex, odds of bystander CPR was significantly higher in communities with higher educational attainment [OR 2 (95 %CI 1.72–2.32) for 96 %-100 % educational attainment compared to ≤ 70 %]. However, in multivariable models adjusting for age and sex, communities with 86 %-90 % and 91 %-95 % had lower odds of bystander CPR [OR 0.84 (95 %CI 0.74–0.95) for 86 %-90 % educational attainment; OR 0.82 (0.72–0.94) for 91 %-95 % educational attainment compared to ≤ 70 %]. Cochran-Armitage trend tests revealed that as the average educational attainment of the community increased, there was a statistically significant increasing trend in performance of bystander CPR (p < 0.001), AED usage (p < 0.001), and obtainment of ROSC (p < 0.001) for POHCA.

This nationwide study of the NEMSIS Database provides substantial support for the association between SDOH and outcomes relating to POHCA. In the largest study of its kind, we confirm that there are statistically significant decreasing trends in the performance of bystander CPR, AED usage, and obtainment of ROSC amongst children suffering OHCA as the minority status and poverty levels of their community increases and community educational attainment decreases. There is mounting scientific evidence establishing that POHCA occurs more frequently amongst children of marginalized communities.^23^, ^31^, ^32^ Identifying and understanding modifiable risk factors amongst these high risk groups, such as barriers to CPR performance and AED usage, is a crucial step in the development of targeted public health interventions to improve outcomes and eliminate health disparities.

Relatively few studies have evaluated associations between SDOH and POHCA outcomes, and the existing literature offers conflicting results.^1^ Some previous studies have pronounced lower performance of bystander CPR,^8^, ^14^, ^33^ less AED usage,^15^ and lower POHCA survival rates^8^, ^9^ for minority children^8^, ^9^, ^14^, ^33^ and those living in communities with higher unemployment and lower income and educational attainment.^15^, ^33^ Other studies, however, have found no such associations.^9^, ^10^, ^11^, ^12^, ^16^, ^17^, ^18^, ^19^ It has been proposed that the variability in findings between these studies may be related to confounding factors, such as location and etiology of the POHCA.^1^ While all previous studies of SDOH and POHCA have been performed amongst significantly smaller sample sizes,^1^, ^8^, ^9^, ^10^, ^11^, ^12^, ^13^, ^14^, ^15^, ^16^, ^17^, ^18^, ^19^, ^20^, ^21^, ^22^, ^23^, ^24^, ^32^, ^33^, ^34^ our study offers a national sample of over 27,000 POHCAs. This much larger sample size would theoretically decrease the impact of these proposed confounders on POHCA outcomes. It has also been proposed that the geographical context and regional variation may influence inconsistencies in previous research findings.^1^ Another related strength of a large, nationwide study is the potential to minimize impacts of geographic variation on POHCA outcomes. The findings of this study in the context of the size and geographic diversity of its sample lends credence to previous literature supporting the associations between community-level SDOH and POHCA outcomes.

While relationships between SDOH and POHCA has been unclear in previous literature, there is strong evidence for effects of sociodemographic disparities in OHCA outcomes amongst adult populations. A variety of studies involving adult OHCA have documented significant associations between socioeconomic disparities and increased incidence of OHCA, lower rates of bystander CPR, and lower survival following OHCA.^7^ It has been postulated that the relationship between socioeconomic disparities and poorer OHCA outcomes amongst adults may be due to chronic comorbidities, such as type two diabetes and congestive heart failure, that tend to develop in adulthood with increased prevalence amongst marginalized populations.^1^ In contrast to adults, chronic disease comorbidity is far less frequently the cause of death amongst children, who are significantly more likely to perish as the result of an acute traumatic event.^35^ Poorer POHCA outcomes amongst marginalized communities in our study is thus less likely to be explained by chronic comorbidity and may be more likely the impact of community-level disadvantage. These findings provide evidence supporting the unique importance of SDOH and their impact on child health outcomes.

When discussing factors impacting POHCA outcomes, it is imperative to consider the most common cause of death amongst children in America: firearm injuries.^36^ In 2020, firearm injuries surpassed motor vehicle accidents as the leading cause of death amongst children, though this increase was not experienced equally throughout different populations.^36^, ^37^, ^38^ Children of racial/ethnic minorities from marginalized communities have suffered the majority of firearm injuries for decades, and this inequity worsened during our study period throughout the COVID19 pandemic.^37^, ^38^, ^39^, ^40^, ^41^ Elevated rates of POHCA amongst marginalized communities may be partially the result of increased child firearm injuries in these areas. Prevention of violent injuries is crucial, and can be accomplished through community-level violence intervention programs.^42^ Targeted interventions to improve POHCA outcomes amongst underserved communities when such injuries occur is equally important to address health disparities and save lives of the children most at risk.

Identification of groups at highest risk for experiencing POHCA and poor POHCA-related outcomes offers the opportunity to implement targeted public health interventions addressing underlying contributing factors. Evidence shows that performance of bystander CPR can be increased by organized CPR training,^43^ and the implementation of CPR training in high-risk communities is one intervention with the potential to improve adult OHCA and POHCA outcomes.^44^ Our study supports the association between lower bystander CPR performance, AED use, and ROSC amongst POHCA occurring in communities with lower rates of completed high-school education. Standardized Basic Life Support (BLS) training amongst schoolchildren of all ages is a germane intervention supported by a recent statement published by the International Liaison Committee on Resuscitation.^44^, ^45^ In order to increase AED usage, training is required in addition to easy public access to AED devises, and some studies suggest decreased AED availability in lower-income areas.^46^, ^47^ Increased AED availability is another modifiable factor with the potential to positively effect POHCA outcomes, though further research on the effects of AED availability on pediatric-specific outcomes is needed.

In addition to community-level factors such as BLS training opportunities and AED availability, a number of city and state-level factors may contribute to inequitable POHCA outcomes. These system-level dynamics can include proximity to hospitals, distribution of ambulances, and urban planning issues impacting traffic flow throughout a city and state.^7^ Some research has indicated that adults experiencing OHCA from economically disadvantaged neighborhoods experience longer EMS times compared to patients from wealthier neighborhoods.^48^ Studies have also found that the COVID19 pandemic, which was still in effect during our study period, had a negative impact on EMS response times, in addition to decreasing rates of bystander CPR and OHCA survival.^49^ Better understanding of these factors as they relate to both adult OHCA and POHCA can inform and improve future public health responses addressing these health disparities.^1^, ^44^

## Limitations

The findings of this study must be interpreted in the context of several limitations. This is a study of nationwide EMS data from the United States of America, and our findings may not be generalizable to all other countries throughout the world*.* Entry of information into the NEMSIS Database is voluntary and may represent a disproportionate number of higher-resourced EMS agencies with the capacity to perform to NEMSIS standards. Thus, the database may not be representative of all EMS agencies.^50^ Data entered into the NEMSIS Database are not audited, and the potential for incomplete or inaccurate figures is a known limitation which can contribute to selection and reporting bias.^50^ Our study included POHCAs of all causes and did not include subgroup analysis of POHCAs based on etiology. There is the potential that POHCA outcomes vary depending on etiology, as well as other factors not included in multivariable analyses, such as the location of the POHCA or who was present when the POHCA occurred. Patient age and sex were adjusted for in multivariable regressions, though there is the potential that other confounding variables may exists that were not adjusted for, such as individual race/ethnicity and comorbidities. While the large sample size of this study has the potential to minimize effects of potential confounding bias, causality still cannot be assumed between associated variables. Additionally, our study assesses for community-level SDOH rather than individual-level SDOH; thus one cannot draw conclusions regarding the impact of an individual patient’s race/ethnicity, poverty level, or educational status on their POHCA outcomes.

## Conclusions

By utilizing a nationwide dataset to perform the largest study yet of SDOH and POHCA outcomes, we establish that there are statistically significant decreasing trends in the performance of bystander CPR, AED usage, and obtainment of ROSC amongst children suffering OHCA as the minority status and poverty levels of their community increases and community educational attainment decreases. Better understanding of the impact of SDOH on POHCA outcomes offers the opportunity to implement public health interventions that can address health disparities and save countless lives.

## Fundings sources/disclosures

All authors declare no conflict of interest related to this work. This research did not receive any specific grants from funding agencies in the public, commercial, or not-for-profit sectors.

## CRediT authorship contribution statement

**Mary E. Bernardin:** Writing – review & editing, Writing – original draft, Supervision, Project administration, Methodology, Investigation, Formal analysis, Data curation, Conceptualization. **Jyoti Arora:** Writing – review & editing, Methodology, Investigation, Formal analysis, Data curation, Conceptualization. **Paul Schuler:** Writing – review & editing, Investigation, Formal analysis, Data curation. **Benjamin Fisher:** Writing – review & editing, Methodology, Investigation, Data curation. **Joseph Finney:** Writing – review & editing, Project administration, Methodology, Investigation, Data curation, Conceptualization. **Elizabeth Kendrick:** Writing – review & editing, Supervision, Project administration, Methodology, Investigation, Data curation, Conceptualization. **Danielle Lee:** Writing – review & editing, Supervision, Project administration, Methodology, Investigation, Data curation, Conceptualization.

## Declaration of competing interest

The authors declare that they have no known competing financial interests or personal relationships that could have appeared to influence the work reported in this paper.
